# Supplementary material for: Methotrexate Alters the Expression of microRNA in Fibroblast-like Synovial Cells in Rheumatoid Arthritis
Source: Int J Mol Sci. 2021 Oct 26;22(21):11561. doi: 10.3390/ijms222111561 (PMC8584010; doi:10.3390/ijms222111561)
Supplement: Supplementary file 1 [file ijms-22-11561-s001.zip › ijms-1431363-supplementary.pdf]

Supplementary Table S1. Multiplex cytokine/chemokine bead assay in the supernatant from RA-FLS transfected with pre-miR-877-3p

	EGF	Eotaxin	FGF-2	FLT-3L	Fractalkine	GM-CSF	IFN-g	IL-10	IL-12p40
Scrambled control	16.39	17.53	58.51	18.7	159.21	3205.3	9.08	14.63	40.37
Pre-miR-887-3p	16.39	18.07	43.77	17.59	132.49	703.47	8.1	10.14	31.89

Supplementary Table S1 (Continued)

IL-12p70	IL-13	IL-15	IL-17	IL-1a	IL-1b	IL-1ra	IL-2	IL-4	IL-7
14.75	18.04	8.58	4.01	8.15	2379.5	26.11	3.87	35.48	26.28
12.58	13.41	7.25	3.92	5.64	3370.1	16.74	3.36	36.76	26.92

Supplementary Table S1 (Continued)

INF-a2	CXCL10	CCL7	CCL22	CCL3	CCL4	TNF-a	TNF-b	VEGF	sCD40L
44.92	272.1	1020.9	28.91	4457.6	384.7	29.32	4.96	185.6	5.81
46.33	97.91	4051.7	25.75	652.6	93	9.89	4.63	178.31	5.27

Values are in pg/ml. Among the measured factors, the values of G-CSF, CXCL1, IL-3, IL-5, IL-6, IL-8, IL-9, CCL2 and TGF-a are not in the table because the concentration was out of range.

EGF epidermal growth factor, *FGF* fibroblast growth factor, *FLT-3L* FMS-like tyrosine kinase 3 ligand, *GM-CSF* granulocyte macrophage colony-stimulating factor, *IFN* interferon, *IL* interleukin, *IL-1ra* interleukin 1 receptor antagonist, *CXCL* C-X-C motif chemokine, *CCL* CC chemokine ligand, *TNF* tumor necrosis factor, *VEGF* vascular endothelial growth factor, *G-CSF* granulocyte colony-stimulating factor, *TGF* transforming growth factor
